# Clinical characteristics and manifestations in older patients with COVID-19

**DOI:** 10.1186/s12877-020-01811-5

**Published:** 2020-10-08

**Authors:** Chenchen Wei, Ya Liu, Yapeng Liu, Kai Zhang, Dezhen Su, Ming Zhong, Xiao Meng

**Affiliations:** 1 The Key Laboratory of Cardiovascular Remodeling and Function Research, Chinese Ministry of Education, Chinese National Health Commission and Chinese Academy of Medical Sciences, The State and Shandong Province Joint Key Laboratory of Translational Cardiovascular Medicine, Department of Cardiology, Qilu Hospital, Cheeloo College of Medicine, Shandong University, Jinan, 250012 China; 2 Department of Gastroenterology, Renmin Hospital of Wuhan University, Wuhan, 430060 China; 3 Department of Infectious Disease, Renmin Hospital of Wuhan University, Wuhan, 430060 China

**Keywords:** SARS-CoV-2, COVID-19, Older patients, Outcome

## Abstract

**Background:**

To investigate the clinical characteristics and manifestations of older patients with coronavirus disease 2019 (COVID-19).

**Methods:**

In this retrospective study, 566 patients with confirmed COVID-19 were enrolled and the clinical characteristics, laboratory findings, complications and outcome data were collected and analyzed.

**Results:**

Among the 566 patients (median age, 61.5 years) with COVID-19, 267 (47.2%) patients were male and 307 (54.2%) were elderly. Compared with younger patients, older patients had more underlying comorbidities and laboratory abnormalities. A higher rate of acute respiratory distress syndrome (ARDS), acute cardiac injury and heart failure was observed in the older group as compared with younger and middle-aged groups, particularly those oldest-old patients had more multi-organ damage. Older patients with COVID-19 were more likely to suffer from acute cardiac injury in cases with preexistenting cardiovascular diseases, while there was no difference among the three groups when patients had no history of cardiovascular diseases. Older patients presented more severe with the mortality of 18.6%, which was higher than that in younger and middle-aged patients (*P* < 0.05). Multivariable analysis showed that age, lymphopenia, ARDS, acute cardiac injury, heart failure and skeletal muscle injury were associated with death in older patients, while glucocorticoids might be harmful.

**Conclusions:**

Older patients, especially the oldest-old patients were more likely to exhibit significant systemic inflammation, pulmonary and extrapulmonary organ damage and a higher mortality. Advanced age, lymphopenia, ARDS, acute cardiac injury, heart failure and skeletal muscle injury were independent predictors of death in older patients with COVID-19 and glucocorticoids should be carefully administered in older patients.

## Background

COVID-19 caused by a novel coronavirus (SARS-CoV-2) has spread rapidly during the past several months. As a novel infectious disease, COVID-19 has the ability of human-to-human transmission and can lead to acute respiratory distress syndrome (ARDS), multiple organ dysfunction and even death [1]. As of April 30, more than three million confirmed cases and more than 200 thousand deaths worldwide were reported by the World Health Organization (WHO).

In China, there were 241 million elderly people aged over 60 years old, among whom more than 30 million were more than 80 years old [2]. The percentage of older adults (≥ 60 years) was 17.3% of the total population. In addition, the number of the aged has dramatically augmented globally and subsequently, the frequency of age-associated diseases, particularly cardiovascular and cerebrovascular diseases, also increased significantly. Although the population is generally susceptible to SARS-CoV-2, older people have higher morbidity and mortality than younger individuals, particularly those with serious underlying comorbidities [3]. The majority of patients infected by SARS-CoV-2 were over 50 years old, and nearly 80% of deaths occurred among individuals aged ≥ 60 years [4].

Considering the complications and age-related vulnerability in older adults, it has been an enormous challenge to protect the function of multi-organ and decrease the mortality from SARS-CoV-2. Although many studies have focused on the clinical characteristics of COVID-19 patients, data about the damage of extrapulmonary organ due to SARS-CoV-2 among the elderly was still limited. Therefore, we aimed to compare the characteristics and manifestations of patients among different age and assess the extrapulmonary organ damage in older patients.

## Methods

### Patients

In this retrospective study, 566 patients with COVID-19 hospitalized from January 3, 2020 to March 16, 2020 at Renmin Hospital of Wuhan University were enrolled. All patients were defined the degree of COVID-19 as moderate or severe according to the Guidance for Corona Virus Disease 2019 (7th edition) issued by the China National Health Commission [5]. The study was approved by the Ethics Committees of Qilu Hospital of Shandong University and Renmin Hospital of Wuhan University.

### Data collection

Epidemiological characteristics, laboratory findings, complications, treatment options and outcomes of enrolled patients were obtained from the electronic medical records. According to recent WHO definitions, patients were categorized into three groups: younger group (< 45 years), middle-aged group (45 to 59 years) and older group (≥ 60 years). People aged 60 years were always still active, whereas individuals aged over 75 years might appear increasingly frail. Therefore, older patients were divided into two groups: the younger old (60 to 74 years) and the oldest-old (≥ 75 years) groups in subgroup analysis.

The occurrence of organ damage was assessed by two independent physicians blinded to the study. ARDS was defined according to the Berlin definition [6]. Acute liver injury was defined as alanine aminotransferase (ALT) and/or aspartate aminotransferase (AST) over 3 × the upper limit unit of normal (ULN), alkaline phosphatase (ALP), gamma-glutamyl transferase (GGT), and/or total bilirubin (TBIL) over 2 × ULN. Acute kidney injury was identified on the basis of the highest serum creatinine level according to the kidney disease improving global outcomes classification [7]. Heart failure was defined by age-related amino-terminal pro-brain natriuretic peptide (NT-proBNP) cut-points (450 pg/mL for patients < 50 years, 900 pg/mL for patients between 50 and 75 years, and 1800 pg/mL for patients > 75 years, respectively) and presence of associated symptoms, including dyspnea and edema. Acute cardiac injury was defined as the serum cardiac troponin I levels (cTnI) above the reference limit (> 0.04 ng/ml). In addition, neurologic manifestations were also assessed, such as nervous system manifestations (dizziness, headache, impaired consciousness and acute cerebrovascular) and skeletal muscular injury manifestations, which indicated a patient had skeletal muscle pain and increased serum levels of creatine kinase.

### Statistical analysis

Data were analyzed using IBM SPSS Statistics for Windows version 25.0 (SPSS Inc. Chicago, IL). The distribution of continuous variables was determined by the Shapiro-Wilk test. Non-normally distributed variables were expressed as median (IQR) and differences between groups were analyzed by the Mann-Whitney U test or Kruskal-Wallis test followed by Dunn’s post hoc. Categorical variables were compared using the χ^2^ test or Fisher’s exact test, as appropriate. Prognostic factors of older patients were identified by univariate and multivariate logistic regression. Only those variables that were identified as significant in the univariate analysis were included in the multivariate analysis. All statistical tests were two-sided, and the differences were considered to be statistically significant at *P* < 0.05.

## Results

### Demographic characteristics among 3 groups

Of 566 patients, the median age was 61.5 years and 47.2% of patients were male. Among the patients, 402 (71.0%) patients met the criteria for severe COVID-19 and 164 (29.0%) patients were moderate cases. There were 102 (18.0%), 157 (27.7%) and 307 (54.2%) patients in younger, middle-aged and older groups, with corresponding median age of 37, 54 and 69 years, respectively. Compared with younger and middle-aged groups, systolic blood pressure and the incidence of any coexisting medical condition were higher in the older group, like the occurrence of hypertension, diabetes and coronary artery disease (CAD). There was no difference of sex and symptoms among the three groups, including the neurologic manifestations (Table 1).

**Table 1:** Demographic and baseline characteristics of COVID-19 patients among 3 groups

	Younger (*n* = 102)	Middle-aged (*n* = 157)	Older (*n* = 307)
Age (years)	37.0 (32.0–41.3)	54.0 (49.5–57.0)	69.0 (65.0–76.0)
Sex			
Male	54 (52.9%)	60 (38.2%)	153 (49.8%)
Female	48 (47.1%)	97 (61.8%)	154 (50.2%)
Typical symptoms			
Fever	71 (69.6%)	126 (80.3%)	248 (80.8%)
Dry cough	63 (61.8%)	108 (68.8%)	183 (59.6%)
Fatigue	38 (37.3%)	76 (48.4%)	144 (46.9%)
Dyspnea	12 (11.8%)	26 (16.6%)	41 (13.4%)
Neurological symptoms			
Dizziness	3/102 (2.9%)	9/156 (5.8%)	16/302 (5.3%)
Headache	5/102 (4.9%)	13/156 (8.3%)	10/302 (3.3%)
Impaired consciousness	0 (0.0%)	0 (0.0%)	3 (1.0%)
Comorbidity			
Any	18 (17.6%)	66 (42.0%)^*^	222 (72.3%)^*#^
Hypertension	6 (5.9%)	52 (33.1%)^*^	158 (51.5%)^*#^
Diabetes	3 (2.9%)	19 (12.1%)^*^	52 (16.9%)^*^
CAD	0 (0.0%)	8 (5.1%)	50 (16.3%)^*#^
Cerebral infarction	0 (0.0%)	1 (0.6%)	21 (6.8%)^*^
Cancer	1 (1.0%)	2 (1.3%)	9 (2.9%)
CKD	2 (2.0%)	1 (0.6%)	7 (2.3%)
COPD	0 (0.0%)	1 (0.6%)	7 (2.3%)
SBP (mmHg)	122.0 (116.0–130.0)	129.0 (120.0–140.0)^*^	134.0 (121.0–148.0)^*#^
DBP (mmHg)	78.0 (70.0–85.0)	80.0 (70.0–87.5)	80.0 (70.0–87.0)
Heart rate (bpm)	85.0 (76.0–98.0)	86.0 (77.5–96.0)	85.0 (77.0–98.0)

Data were presented as n (%) or median (IQR). ^*^P < 0.05 vs younger group; ^#^*P* < 0.05 vs middle-aged group. *CAD* coronary artery disease, *CKD* chronic kidney disease, *COPD* chronic obstructive pulmonary disease, *SBP* systolic blood pressure, *DBP* diastolic blood pressure

### Laboratory findings among 3 groups

When compared with younger and middle-aged groups, older patients exhibited higher counts of neutrophils and serum levels of C-reactive protein (CRP), AST, LDH, blood glucose, urea nitrogen and creatinine as well as lower numbers of lymphocytes, hemoglobin and platelet (*P* < 0.05). More severe lymphopenia occurred in older individuals where 101 (32.9%) patients had lymphocyte counts below 0.8 × 10^9^/L, while only 13 (12.7%) and 29 (18.5%) in younger and middle-aged groups, respectively (Table 2).

**Table 2:** The laboratory findings, complications, treatment and clinical outcome of patients with COVID-19 among 3 groups

	Younger (*n* = 102)	Middle-aged (*n* = 157)	Older (*n* = 307)
White blood cells (10^9^/L)	5.57 (4.16–7.18)	5.37 (4.28–7.07)	5.73 (4.53–7.56)
Neutrophils (10^9^/L)	2.93 (2.11–4.66)	3.14 (2.55–4.59)	3.87 (2.73–5.56)^*#^
Lymphocytes (10^9^/L)	1.52 (1.09–2.02)	1.31 (0.90–1.73)	1.01 (0.68–1.57)^*#^
< 0.8	13 (12.7%)	29 (18.5%)	101 (32.9%)^*#^
Hemoglobin (g/L)	133.0 (122.8–145.0)	125.0 (116.5–135.5)^*^	121.0 (111.0–132.0)^*#^
Platelet (10^9^/L)	220.5 (177.3–272.3)	231.0 (178.0–295.5)	208.0 (155.0–261.0)^#^
< 100	0 (0.0%)	2 (1.3%)	19 (6.2%)^*#^
CRP (mg/L)	2.53 (0.50–11.88)	5.60 (0.71–37.40)	27.70 (2.64–72.85)^*#^
ALT (U/L)	26.0 (17.8–48.0)	27.0 (18.0–51.0)	23.0 (16.0–36.0)^#^
AST (U/L)	23.0 (17.0–33.3)	25.0 (19.0–37.0)	27.0 (19.0–39.0)^*^
ALP (U/L)	57.0 (45.0–69.3)	64.5 (52.0–81.3)^*^	66.0 (55.0–81.0)^*^
GGT (U/L)	23.0 (15.0–39.3)	29.5 (18.0–51.0)	27.0 (17.0–47.0)
Total bilirubin (μmol/L)	10.1 (7.2–13.0)	10.2 (7.5–13.7)	11.1 (8.4–15.2)^*#^
Albumin (g/L)	41.5 (38.4–44.7)	39.4 (36.6–42.1)^*^	36.6 (33.1–39.3)^*#^
< 30	0 (0.0%)	0 (0.0%)	18 (5.9%)^*#^
Urea nitrogen (mmol/L)	4.11 (3.10–5.42)	4.53 (3.40–5.61)	5.40 (4.28–7.41)^*#^
Creatinine (μmol/L)	61.0 (48.8–72.0)	55.0 (46.5–67.5)	63.0 (52.0–75.0)^#^
Blood glucose (mmol/L)	4.99 (4.59–5.87)	5.36 (4.83–6.76)^*^	5.80 (5.04–7.43)^*#^
Creatine kinase (U/L)	61.0 (44.0–108.0)	56.0 (35.0–82.5)	60.0 (42.0–94.5)
LDH (U/L)	189.0 (164.8–298.5)	227.0 (187.0–281.0)	265.0 (210.0–363.0)^*#^
CK-MB (ng/mL)	0.64 (0.44–0.88)	0.75 (0.54–1.05)	1.27 (0.90–2.25)^*#^
cTnI (ng/mL)	0.006 (0.006–0.006)	0.006 (0.006–0.006)	0.008 (0.006–0.025)^*#^
Complications			
ARDS	15 (14.7%)	35 (22.3%)	88 (28.7%)^*^
Acute cardiac injury	4/91 (4.4%)	5/154 (3.2%)	66/306 (21.6%)^*#^
With history of cardiovascular disease	0/5 (0.0%)	0/53 (0.0%)	56/178 (31.5%)^#^
Without history of cardiovascular disease	4/86 (4.7%)	5/101 (5.0%)	10/128 (7.8%)
Heart failure (elevated BNP)	1/72 (1.4%)	5/132 (3.8%)	39/269 (14.5%)^*#^
Novel or worsening arrhythmia	21 (20.6%)	26 (16.6%)	71 (23.1%)
Acute liver injury	9 (8.8%)	11 (7.0%)	35 (11.4%)
Acute kidney injury	2 (2.0%)	1 (0.6%)	13 (4.2%)
Skeletal muscle injury	9/102 (8.8%)	8/150 (5.3%)	20/285 (7.0%)
Treatment			
Antiviral therapy	97 (95.1%)	150 (95.5%)	295 (96.1%)
Antibiotic therapy	66 (64.7%)	114 (72.6%)	230 (74.9%)
Glucocorticoids	27 (26.5%)	42 (26.8%)	127 (41.4%)^*#^
Oxygen support			
Nasal cannula	71 (69.6%)	111 (70.7%)	220 (71.7%)
Non-invasive ventilation (ie, face mask)	9 (8.8%)	25 (15.9%)	65 (21.2%)^*^
Invasive mechanical ventilation	1 (1.0%)	4 (2.5%)	12 (3.9%)
Severe	49 (48.0%)	99 (63.1%)^*^	254 (82.7%)^*#^
Clinical outcome			
Discharge	100 (98.0%)	150 (95.5%)	250 (81.4%)^*#^
Death	2 (2.0%)	7 (4.5%)	57 (18.6%)^*#^

^*^P < 0.05 vs younger group; ^#^*P* < 0.05 vs middle-aged group. *CRP* C-reactive protein, *ALT* alanine aminotransferase, *AST* aspartate aminotransferase, *ALP* alkaline phosphatase, *GGT* gamma-glutamyl transferase, *LDH* lactate dehydrogenase, *CK-MB* creatine kinase-myocardial isoenzyme, *cTnI* cardiac troponin *I* ARDS acute respiratory distress syndrome

Common complications were assessed and we observed that 28.7% of older patients suffered the ARDS, while the proportion of individuals exhibiting ARDS in younger and middle-aged patients were 14.7% and 22.3%, respectively. Elevated cTnI and BNP levels were found in the older group when compared with those in younger and middle-aged groups, suggesting a higher rate of acute cardiac injury and heart failure. Surprisingly, older patients with preexisting cardiac comorbidity were more likely to suffered cardiac injury, while there was no difference of the incidence of acute cardiac injury among patients without cardiovascular diseases. On admission, there was decreased level of albumin in the older group with 18 (5.9%) older patients developing hypoalbuminemia (albumin < 30 g/L), suggesting a poor nutrition status of older patients. There was no difference of novel or worsening arrhythmia, acute liver injury, acute kidney injury and skeletal muscle injury among the three groups (Table 2).

Among the total of 566 patients, 95.8% of individuals received antiviral agents (including arbidol, ribavirin, oseltamivir and ganciclovir) and more than half of patients received antibacterial therapy. 34.6% of patients received glucocorticoids therapy (methylprednisolone, 40-80 mg/day for 5–7 days). More glucocorticoids were used in older patients (41.4%) than younger and middle-aged patients (26.5% or 26.8%, *P* < 0.05). 82.7% of older patients were severe and 18.6% of them died eventually, which was higher than that in younger and middle-aged patients (*P* < 0.05). There was no significant difference of treatment and mortality between younger and middle-aged groups (Table 2).

### The subgroup analysis of older patients

In subgroup analysis, older patients were further divided into two groups: younger old and oldest-old groups. Among the 307 older patients, there were 216 (70.4%) younger old patients and 91 (29.6%) oldest-old patients. The incidence of hypertension and CAD was more frequent in oldest-old patients. Compared with the younger old, oldest-old patients had higher counts of neutrophils and serum levels of CRP, AST, urea nitrogen, creatinine, LDH, CK-MB and cTnI, while lower counts of lymphocytes and platelet and serum albumin levels (*P* < 0.05). Oldest-old patients exhibited higher incidence of extrapulmonary organ damage, including acute cardiac injury, heart failure, skeletal muscle injury and kidney injury than those in younger old patients (*P* < 0.05). Importantly, the mortality of the oldest-old patients was much higher than younger old patients (*P* < 0.001, Table 3).

**Table 3:** The laboratory findings, complications, treatment and clinical outcome in the older patients

	Younger old (*n* = 216)	Oldest-old (*n* = 91)	*P*
Age (years)	66.0 (63.0–69.0)	79.0 (76.0–82.0)	< 0.001
Male	96 (44.4%)	57 (62.6%)	0.004
Comorbidity			
Any	143 (66.2%)	79 (86.8%)	< 0.001
Hypertension	96 (44.4%)	62 (68.1%)	< 0.001
Diabetes	37 (17.1%)	15 (16.5%)	0.890
CAD	27 (12.5%)	23 (25.3%)	0.006
Cerebral infarction	8 (3.7%)	13 (14.3%)	0.001
Cancer	6 (2.8%)	3 (3.3%)	1.000
CKD	3 (1.4%)	4 (4.4%)	0.233
COPD	3 (1.4%)	4 (4.4%)	0.233
Laboratory findings			
White blood cells (10^9^/L)	5.72 (4.34–7.39)	5.96 (4.74–8.12)	0.155
Neutrophils (10^9^/L)	3.70 (2.54–5.47)	4.30 (3.37–6.54)	0.006
Lymphocytes (10^9^/L)	1.18 (0.73–1.68)	0.85 (0.59–1.29)	< 0.001
< 0.8	58 (26.9%)	43 (47.3%)	0.001
Hemoglobin (g/L)	121.0 (112.0–131.0)	121.0 (109.0–133.0)	0.660
Platelet (10^9^/L)	213.0 (165.3–269.5)	197.0 (134.0–242.0)	0.011
< 100	10 (4.6%)	9 (9.9%)	0.081
CRP (mg/L)	18.90 (1.37–73.09)	53.55 (12.28–73.03)	0.001
ALT (U/L)	24.0 (16.0–35.8)	23.0 (16.0–39.0)	0.829
AST (U/L)	25.0 (18.0–35.8)	30.0 (21.0–48.0)	0.002
Albumin (g/L)	37.1 (33.6–40.0)	35.0 (32.0–37.7)	< 0.001
< 30	11 (5.1%)	7 (7.7%)	0.376
Urea nitrogen (mmol/L)	5.10 (4.00–6.51)	7.70 (4.80–11.31)	< 0.001
Creatinine (μmol/L)	60.0 (50.0–70.0)	68.0 (57.0–100.0)	< 0.001
Blood glucose (mmol/L)	5.80 (5.06–7.74)	5.79 (4.90–7.01)	0.658
Creatine kinase (U/L)	59.0 (42.0–86.5)	62.0 (42.5–150.0)	0.103
LDH (U/L)	252.0 (200.0–346.0)	289.0 (230.0–452.0)	0.001
CK-MB (ng/mL)	1.12 (0.83–1.63)	2.27 (1.32–4.43)	< 0.001
cTnI (ng/mL)	0.006 (0.006–0.014)	0.027 (0.010–0.193)	< 0.001
Complications			
ARDS	56 (25.9%)	32 (35.2%)	0.102
Acute cardiac injury	23/215 (10.7%)	43/91 (47.3%)	< 0.001
With history of cardiovascular disease	19/108 (17.6%)	37/70 (52.9%)	< 0.001
Without history of cardiovascular disease	4/107 (3.7%)	6/21 (28.6%)	0.001
Heart failure (elevated BNP)	21/185 (11.4%)	18/84 (21.4%)	0.030
New or worsening arrhythmia	54 (25.0%)	17 (18.7%)	0.230
Acute liver injury	20 (9.3%)	15 (16.5%)	0.069
Acute kidney injury	4 (1.9%)	9 (9.9%)	0.004
Skeletal muscle injury	9/204 (4.4%)	11/81 (13.6%)	0.006
Clinical outcome			
Discharge	191 (88.4%)	59 (64.8%)	< 0.001
Death	25 (11.6%)	32 (35.2%)	

In addition, the older patients were divided into two sub-groups: survivors (*n* = 250) and non-survivors (*n* = 57). Compared with survivors, non-survivors were older, and had higher comorbidity rates including hypertension and CAD (*P* < 0.05). Non-survivors were more likely to suffer ARDS (68.4% vs. 19.6%), acute myocardial injury (71.9% vs. 10.0%), heart failure (43.4% vs. 7.4%), skeletal muscle injury (26.7% vs. 3.3%) and acute kidney injury (10.5% vs. 2.8%, all *P* < 0.05) as compared with survivors. Among all 57 non-survivors, 53 patients (93.0%) had ARDS and/or cardiac complications (Table S1).

### The predictors for the prognosis of elderly patients

Univariate and multivariable logistic regression analyses were performed and we found that age was an independent risk factor for the prognosis of older COVID-19 patients, and the risk of death increased by 8.5% per year approximately in elderly population. ARDS, acute cardiac injury, heart failure, skeletal muscle injury and lymphopenia were all independent risk factors associated with death. Disappointedly, glucocorticoids were associated with an increased mortality in older patients with COVID-19 (OR: 3.990; 95%CI: 1.364–11.668; *P* = 0.011, Table 4).

**Table 4:** Univariate and multivariate regression analysis for predicting the risk of death in older patients with COVID-19

	Univariate		Multivariate	
	OR (95%CI)	*P*	OR (95%CI)	*P*
Age (years)	1.111 (1.069–1.155)	0.000	1.085 (1.017–1.158)	0.014
History of cardiovascular disease	3.299 (1.665–6.535)	0.001		
ARDS	8.888 (4.687–16.853)	0.000	8.576 (3.084–23.850)	0.000
Acute cardiac injury	22.960 (11.284–46.717)	0.000	3.690 (1.233–11.042)	0.020
Heart failure	9.583 (4.552–20.178)	0.000	4.782 (1.543–14.817)	0.007
New or worsening arrhythmia	1.384 (0.722–2.652)	0.328		
Acute liver injury	1.915 (0.862–4.253)	0.111		
Acute kidney injury	4.084 (1.317–12.661)	0.015		
Skeletal muscle injury	10.545 (4.013–27.709)	0.000	7.330 (1.453–36.977)	0.016
Leukocytosis	10.635 (4.897–23.094)	0.000		
Lymphopenia	4.435 (2.427–8.105)	0.000	2.793 (1.030–7.578)	0.044
Hypoalbuminemia	3.042 (1.124–8.231)	0.028		
Antiviral therapy	1.146 (0.244–5.378)	0.863		
Antibiotic therapy	7.714 (2.338–25.450)	0.001		
Glucocorticoids	3.989 (2.155–7.384)	0.000	3.990 (1.364–11.668)	0.011

## Discussion

SARS-CoV-2 was more likely to affect older patients, particularly those with comorbidities [3]. To our best knowledge, this is the first report to systematically describe the extrapulmonary organ damage caused by SARS-CoV-2 in older patients. In this study, we showed that older patients with COVID-19 exhibited more coexisting diseases, ARDS and extrapulmonary organ damage, which were associated with a higher mortality. Moreover, oldest-old patients showed higher incidence of multi-organ dysfunction and mortality.

Aging is a complex and multifactorial process. Frailty, the common problem of aging population, is a nonspecific state of vulnerability to poor resolution of homeostasis following a stress [8]. The prevalence of frailty increased with age, the elderly older than age 65 accounted for 22.4%, while 43.7% for those over 85 years old [9]. Increasing frailty with age was associated with increased risk of infection and mortality [4]. In this study, 41.7% of patients were aged over 65 years and 3.2% was 85 or even older. In addition, many older patients with COVID-19 had one or more comorbities, making them more susceptible to SARS-CoV-2 infection and had greater severe illness. In this study, 72.3% of older patients had at least one coexisting illness.

Compared with SARS, COVID-19 had a lower case fatality. It has been reported that the over-all mortality was 1.4% [10]. Although most younger people infected by SARS-CoV-2 were asymptomatic or mildly ill, the elderly exhibited more severe symptoms and higher mortality. It is estimated that the mortality was 1.4% in the elderly less than 60 years old while increased by 4.5% in older individuals over 60 years old and by 14.8% in patients aged 80 or older [11]. In this study, the condition of older patients was more serious and the mortality was 18.6%, which was significantly higher than that of younger and middle-aged patients.

SARS-CoV-2 infection is able to result in clusters of severe pneumonia and even ARDS, which is the leading cause of death in patients with COVID-19. In one study enrolling 201 COVID-19 patients, 41.8% of patients developed ARDS and 52.4% of them died eventually [12]. In another study by Lian J et al., 16.9% of older patients (≥60 years) developed ARDS, which only accounted for 5.37% in younger patients (< 60 years) [13]. They argued that older age was associated with greater risk of development of ARDS and death [13]. In this study, older patients exhibited higher counts of neutrophils and CRP levels, suggesting an augmented inflammatory response. Meanwhile, older patients showed a higher incidence of ARDS, which was an independent risk factor for death.

Some COVID-19 patients without common symptom (fever or cough) came to hospital with only cardiovascular manifestations as their presenting symptoms. Cardiac injury is also one of essential causes of death in patients with COVID-19. Patients with cardiovascular comorbidities were more likely to develop cardiac complications and Ruan Q et al. reported that 40% of deaths were associated with circulatory failure due to cardiac injury [14]. In this study, older patients had increased incidence of acute cardiac injury and heart failure, which were independently associated with poor outcome in elderly patients with COVID-19. Importantly, acute cardiac injury could be developed no matter whether there was cardiovascular disease previously.

Nutritional status of the host exerts a crucial role in the defense against infection, and individuals with nutritional deficiency are more susceptible to a series of infectious diseases which can lead to a detrimental consequence [15, 16]. Malnutrition has been considered as an independent risk factor for increased complications and higher mortality in hospitalized patients [17]. The basic nutritional status of older patients with chronic diseases is always poor, which makes them tend to be critically ill after infection [18]. In this study, the older patients were more likely to develop hypoalbuminemia and decreased hemoglobin, suggesting they were under a poor nutrition state, which might be a cause of higher mortality in older patients.

Recently, a descriptive study indicated 36.4% of patients with COVID-19 had nervous system manifestations [19]. However, we did not find any difference of nervous system manifestations among the three groups. And, we also did not find any difference of acute liver or kidney injury. However, oldest-old patients showed higher skeletal muscle injury and acute kidney injury.

Immune system exerts a central role in host-viral interactions and aging has the ability to induce a series of changes that affect the immune system. Immunosenescence is an age-related process that affects both innate and adaptive immunity, increasing the vulnerability and mortality of elderly to infectious diseases [20]. Immune organs such as thymus and lymph nodes atrophy gradually with age, and aging has a profound impact on the phenotype and functions of various immune cells [20, 21]. Many patients with COVID-19 exhibited lymphopenia, which was more prominent in those severe patients [10, 22]. In our study, persistent and more severe lymphopenia was observed in older patients, 32.9% of whom had lymphocyte counts below 0.8 × 10^9^/L, which suggested a damaged immune system. The damaged immune function, caused by immunosenescence and preceding coronavirus infection, increased the susceptibility to secondary bacterial pneumonia.

To date, no vaccine or specific therapeutic drug for COVID-19 has been approved. The treatment of elderly patients faces special challenges due to complications and general age-related vulnerability. Although glucocorticoids treatment was likely required in patients with coronavirus pneumonia, their efficacy remains controversial. A meta-analysis enrolled 5270 patients showed that corticosteroid use may increase mortality and serious adverse reactions in patients with COVID-19 [23]. However, in RECOVERY trial, dexamethasone (6 mg once daily for up to 10 days) treatment reduced 28-day mortality among those COVID-19 patients receiving invasive mechanical ventilation or oxygen at randomization but not among patients receiving no respiratory support [24]. Among 2104 patients, 54% of patients with dexamethasone therapy were younger than 70 years [24]. Older patients always have more comorbidities and they are not sensitive to many drugs and have more adverse effects of prescription drugs than younger patients. Therefore, glucocorticoids use may present a different effect in older patients with COVID-19. In our study, we focused on the effect of glucocorticoids in the older patients with COVID-19 and found glucocorticoids use were associated with an increased mortality in older patients. Maybe the different results were due to the heterogeneity in the choice, dosage and timing of steroids therapy, different crowds, medical conditions and disease severity. Therefore, caution in glucocorticoids use in older patients is advisable.

There were several limitations in this study. First of all, this was a single-center, retrospective analysis and a number of confounding factors may influence the clinical outcomes. Secondly, as one of the designated hospitals for severe COVID-19, most of patients enrolled were severe cases, which might be different from the whole infected population.

## Conclusions

As the host receptor of SARS-CoV-2, ACE2 exists in multiple human organs, including lung, heart, nervous system and skeletal muscles, which may explain why SARS-CoV-2 is able to lead to multiple organs damage. Older patients, especially the oldest-old patients are more likely to exhibit significant systemic inflammation, pulmonary and extrapulmonary organ damage and a higher mortality. This may be due to more comorbidities, immunosenescence, decreased immune function, poor nutrition state and increased frailty in older patients. Age, ARDS, acute cardiac injury, heart failure, skeletal muscle injury and lymphopenia are associated with the mortality and glucocorticoids may be harmful for older patients with COVID-19. Considering the higher severity and lethality in older patients with COVID-19, physicians should closely monitor and prevent the possible organ damage to improve their survival and reduce the mortality.

## Supplementary information

**Additional file 1.**

## Data Availability

The datasets used during the current study are available from the corresponding author on reasonable request.

## Abbreviations

ALP: Alkaline phosphatase
ALT: Alanine aminotransferase
ARDS: Acute respiratory distress syndrome
AST: Aspartate aminotransferase
CAD: Coronary artery disease
CK-MB: Creatine kinase-myocardial isoenzyme
COVID-19: Coronavirus disease 2019
CRP: C-reactive protein
cTnI: Cardiac troponin I
GGT: Gamma-glutamyl transferase
LDH: Lactate dehydrogenase
NT-proBNP: Amino-terminal pro-brain natriuretic peptide
TBIL: Total bilirubin
