# Supplementary material for: Clinical characteristics and manifestations in older patients with COVID-19
Source: BMC Geriatr. 2020 Oct 8;20:395. doi: 10.1186/s12877-020-01811-5 (PMC7542569; doi:10.1186/s12877-020-01811-5)
Supplement: Supplementary file 1 — Additional file 1. [file 12877_2020_1811_MOESM1_ESM.docx]

**Table S1. The baseline characteristics and complications in the older patients.**

|  | Survivors (n=250) | Non-survivors (n=57) | *P* |
| --- | --- | --- | --- |
| Age (years) | 68.0 (64.0-74.0) | 76.0 (69.0-80.5) | <0.001 |
| Male | 121 (48.4%) | 32 (56.1%) | 0.292 |
| Comorbidity | | | |
| Any | 172 (68.8%) | 50 (87.7%) | 0.004 |
| Hypertension | 120 (48.0%) | 38 (66.7%) | 0.011 |
| Diabetes | 40 (16.0%) | 12 (21.1%) | 0.359 |
| CAD | 32 (12.8%) | 18 (31.6%) | 0.001 |
| Cerebral infarction | 14 (5.6%) | 7 (12.3%) | 0.130 |
| Cancer | 7 (2.8%) | 2 (3.5%) | 1.000 |
| CKD | 5 (2.0%) | 2 (3.5%) | 0.844 |
| COPD | 6 (2.4%) | 1 (1.8%) | 1.000 |
| Complications | | | |
| ARDS | 49 (19.6%) | 39 (68.4%) | <0.001 |
| Acute cardiac injury | 25/249 (10.0%) | 41/57 (71.9%) | <0.001 |
| Heart failure | 16/216 (7.4%) | 23/53 (43.4%) | <0.001 |
| New or worsening arrhythmia | 55 (22.0%) | 16 (28.1%) | 0.327 |
| Acute liver injury | 25 (10.0%) | 10 (17.5%) | 0.106 |
| Acute kidney injury | 7 (2.8%) | 6 (10.5%) | 0.024 |
| Skeletal muscle injury | 8/240 (3.3%) | 12/45 (26.7%) | <0.001 |
